# Impact of air quality policy scenarios on pollution inequalities in a UK metropolitan area

**DOI:** 10.3389/fpubh.2025.1690825

**Published:** 2025-10-13

**Authors:** James R. Hodgson, Suzanne Bartington, Zongbo Shi, William J. Bloss

**Affiliations:** ^1^Institute of Applied Health Research, University of Birmingham, Birmingham, United Kingdom; ^2^School of Geography, Earth and Environmental Sciences, University of Birmingham, Birmingham, United Kingdom

**Keywords:** PM_2.5_, air quality, inequalities, population exposure, policy objectives

## Abstract

Fine particles (particulate matter (PM)_2.5_) affect health, with no observable thresholds below which exposure can be considered safe. The World Health Organization (WHO) Air Quality Guidelines (AQGs, 2021) reflect this evidence base, providing ambitious, health-based guidelines and interim targets for the protection of human health worldwide. In England, the Environment Act 2021 established new, legally binding environmental targets for long-term PM_2.5_ concentrations, comprising both an annual mean concentration target and a population exposure reduction target (PERT), to be achieved by 31 December 2040. However, the benefits of these targets in reducing air pollution inequalities among different sociodemographic groups remain undefined. Using the West Midlands Combined Authority (WMCA, population ~2.9 million) as a reference, we assessed the health impacts of ambient PM_2.5_ concentrations in the year 2019 and evaluated the extent to which existing UK targets and the WHO guidelines address air quality inequalities in this diverse, metropolitan region. We found that ~41% of the WMCA population lived in areas where annual mean PM_2.5_ concentrations exceeded the long-term annual mean concentration target of 10 μg m^−3^. In addition, PM_2.5_ levels across the region exceeded the WHO AQGs. PM_2.5_ concentrations were significantly higher for those living in areas with greater socioeconomic deprivation. The WMCA has a younger-than-average population, which further increases health risks for residents. The most significant health benefits are experienced in the most deprived and densely populated areas. The PERT approach offers the broadest population-level benefits across the region; however, it may still leave some of the most deprived locations failing to meet the AQG targets. Our findings suggest that coordinated regional actions to improve air quality, in line with PERT approaches and pathways towards achieving the WHO guidelines, will deliver the greatest impact on reducing health inequities in the region.

## 1 Introduction and background

Ambient air pollution is internationally recognised as the largest environmental risk to public health, contributing to ~4.2 million premature deaths globally each year (1). In England, air pollution is responsible for an estimated 26,000–38,000 premature deaths per year, equivalent to a reduction in life expectancy of up to 6 months (2–5). In addition, short- and long-term exposure to air pollution contributes to adverse health outcomes and exerts direct and indirect economic costs on regional and national economies (45). The three main pollutants responsible for health harms are particulate matter (PM), nitrogen dioxide (NO_2_), and ozone (O_3_), with the most consistent epidemiological evidence for long-term mortality and chronic disease morbidity burden associated with exposure to fine PM (PM_2.5_) (6–8).

In autumn 2021, the World Health Organization (9) updated its 2005 Global Air Quality Guidelines (AQGs), reflecting advances in scientific evidence from large-scale epidemiological studies that demonstrate adverse health impacts at lower pollutant exposure levels (Table 1). In addition to the AQGs, the WHO provided interim targets, designed as a framework for stepwise progress to support continuous air quality improvement (9). It is important to note that the WHO guideline values reflect the lowest level at which health harms have been detected in large-scale studies. However, evidence suggests no lower threshold; therefore, efforts should be made to reduce PM_2.5_ levels as much as possible (10–12). Furthermore, the WHO AQGs are not legally binding recommendations but are intended to be transferred into practicable standards for the protection of human health.

**Table 1:** Selected 2040 air quality objectives for England and the updated 2021 WHO guideline levels.

Pollutant	Averaging time	Air quality objective (England)	WHO 2021 update				
			Interim targets				Guidelines
Fine particulates (PM_2.5_)	Annual mean	10	**1**	**2**	**3**	**4**	5
			35	25	15	10	

Concentrations are in μg m^−3^.

The UK government published its Environmental Targets (Fine Particulate Matter) Regulations on 30 January 2023, including the legal adoption of a long-term annual mean PM_2.5_ concentration-based target (<10 μg m^−3^) and a population exposure reduction target (PERT) of a 35% reduction (compared to 2018 levels)—both to be achieved across England by 2040 (13). The inclusion of an exposure reduction target provides an opportunity to shift the emphasis of air quality mitigation actions away from those that target pollutant concentration reduction at specific locations towards wider interventions that may benefit a larger population, particularly in areas with high population density.

The health impacts of poor air quality are unevenly distributed between localities, with vulnerable and susceptible populations disproportionately affected. Population density, demography, and health geography of the affected population are all relevant for determining overall health effects at a population level (14–18). However, the most socio-economically disadvantaged communities typically experience higher rates of air pollution-related morbidity and mortality compared to more affluent populations and may also lack the capability or agency to make changes to their lifestyles or residential locations to reduce exposure (19–21). It is also important to note that air quality is only one of the wider determinants of health; the social-ecological systems in which people live, work, and spend their leisure time are also factors (14, 16, 17). Importantly, these factors largely determine health disparities across the UK and are collectively estimated to account for approximately 80% of an individual’s long-term health outcomes. Quantification of the health benefits achieved by “limit-value” concentration targets and exposure reduction approaches to air quality improvement is required to understand which interventions will deliver the greatest societal benefit (22) and reduce air pollution inequalities. Consequently, using routine data sources, we investigated the relative impact of achieving UK air quality targets and the WHO guidelines and assessed the benefits of reducing spatial differences in air pollution inequalities among residents living in an urban metropolitan region.

### 1.1 Setting

The study location was the West Midlands Combined Authority (WMCA) region, which consists of seven local authority metropolitan areas in central England: Birmingham, Coventry, Dudley, Sandwell, Solihull, Walsall and Wolverhampton, with an ethnically diverse population of ~2.9 million in 2019 (23). The WMCA has a committed vision to deliver a carbon-neutral region by 2041 (WM2041). Key air pollutants of health concern are nitrogen dioxide (NO_2_) and fine PM_2.5_. In the West Midlands, up to 2,300 premature deaths were estimated to be attributable to PM_2.5_ pollution in 2019, with the highest mortality burden occurring in Birmingham and Sandwell (24). Until recently, most regional policy efforts have focused on achieving legal compliance with air quality objectives, primarily roadside NO_2_ exceedances of legal limit values in defined areas. This focus has led to local interventions such as the Birmingham Clean Air Zone, introduced in June 2021. More recently, in 2023, the WMCA adopted the Air Quality Framework that aims to ‘reduce absolute and unequal exposure to poor air quality giving everyone better air to breathe and improving health outcomes’ (25, 44). Importantly, the framework includes a greater focus on addressing PM_2.5_ pollution due to the increased policy attention and adverse health effects of the pollutant. Furthermore, actions to reduce PM_2.5_ exposure among populations who experience the greatest existing air pollution-related burden of disease are also examined in the framework.

## 2 Data sources

### 2.1 Air quality data

Air quality data were obtained from Defra’s background air quality PM_2.5_ concentration map (2019) (42, 43). This consists of the mean estimated annual average PM_2.5_ concentration for 2019 assigned to 1 km x 1 km grid squares across the West Midlands. These grid data were interpolated using ordinary kriging within a GIS to generate a midlands-wide PM_2.5_ map. These interpolated data were then extracted at the ward, local authority and regional levels for the WMCA area, providing the mean value for each ward and local authority and across the WMCA. Defra background air quality data were utilised to enable UK-wide replication.

### 2.2 Demographic and socio-economic deprivation data

Population age structure and Index of Multiple Deprivation (IMD) data for all 192 WMCA wards were obtained from the Office for National Statistics (ONS) and the Ministry of Housing, Communities & Local Government, respectively (23, 26) for 2019. Population data are shown below in Table 2, and it is worth noting that, on average, the WMCA population is younger than the national average for England.

**Table 2:** Demographic information for the seven constituent local authorities within the WMCA (23).

Population	Birmingham	Coventry	Dudley	Sandwell	Solihull	Walsall	Wolverhampton	Total
0–17 years	287,393	79,765	69,644	82,449	47,549	68,970	62,276	698,046
18–29 years	238,971	91,752	43,724	49,244	27,610	42,053	40,151	533,505
30–39 years	158,926	55,240	40,252	48,429	25,210	38,200	37,027	403,284
40–49 years	133,683	40,508	39,924	41,642	27,191	34,752	33,307	351,007
50–59 years	124,958	38,576	44,681	41,378	31,045	34,532	33,485	348,655
60–69 years	89,974	28,799	34,789	29,297	23,404	27,613	25,087	258,963
70–79 years	63,766	22,620	30,420	22,246	20,957	22,715	18,029	200,753
80 < years	44,145	14,261	18,262	13,765	13,408	14,361	12,416	130,618
Total	1,141,816	371,521	321,596	328,450	216,374	285,478	263,357	2,928,592

Source: ONS (23).

The IMD is an official composite measure of relative deprivation for area locations across England, ranking them from the most deprived to the least deprived (26). It is calculated using seven weighted domains: income deprivation; employment deprivation; education, skills, and training deprivation; health deprivation and disability; crime; barriers to housing and services; and living environment deprivation (26). Wards were classified into deciles using the IMD score. It should be noted that these deciles are specific to the WMCA and not for the overall population.

### 2.3 Estimation of population PM_2.5_ exposure

To estimate PM_2.5_ exposure at the ward level, we calculated the population-weighted exposure level (PWEL), which accounts for both the population living in a geographical area and the PM_2.5_ concentration to which they are exposed (27–31). This method was used to produce an area-specific population-weighted average concentration. To calculate the PWEL (equation 1), an area’s (e.g., Lower Super Output Area, ward, or local authority) population is multiplied by its mean PM_2.5_ value and then divided by the total population of the wider region (e.g., county or region). In this study, the PWEL was applied at the ward level across the WMCA region (total population):


(1) $$\text{PWEL}\;(\mathrm{\mu g}/m^{3})=\frac{\begin{array}{l} \;\text{Study Area average}\;{\mathrm{PM}}_{2.5}\;\text{concentration}\\ \times \text{Study Area}\;(\text{ward})\;\text{Population} \end{array}}{\text{Total Population}\;(\text{WMCA})}$$

The PWEL calculation utilised in this study (Equation 1).

### 2.4 Air quality scenarios

Three air quality policy scenarios were examined to determine how changes in annual average PM_2.5_ concentrations (at the ward level) in the WMCA area would influence population air pollution exposure compared to 2019 levels:

Scenario A: “Limit Value”: Attainment of the 10 μg/m^3^ target.
Scenario B: “PERT”: A population exposure reduction of 35% in PM_2.5_ concentrations compared to 2019.
Scenario C: “WHO”: Attainment of the WHO AQGs for PM_2.5_ (5 μgm^3^) in all wards.

The scenarios were selected as they are most relevant to existing UK PM_2.5_ targets and health-based guidelines, and therefore reflect the current focus of air quality policy efforts. The analysis was intended to quantify the benefits that would be achieved if such changes in PM_2.5_ levels were implemented. In this study, we did not examine the feasibility or delivery mechanisms of associated policies.

### 2.5 Data linkage and statistical analysis

All mapping, scenarios, and data analysis were performed using Esri ArcMap and R Studio, with data linkage conducted using ward code data.

## 3 Results

### 3.1 Regional air quality

**Figure 1:**
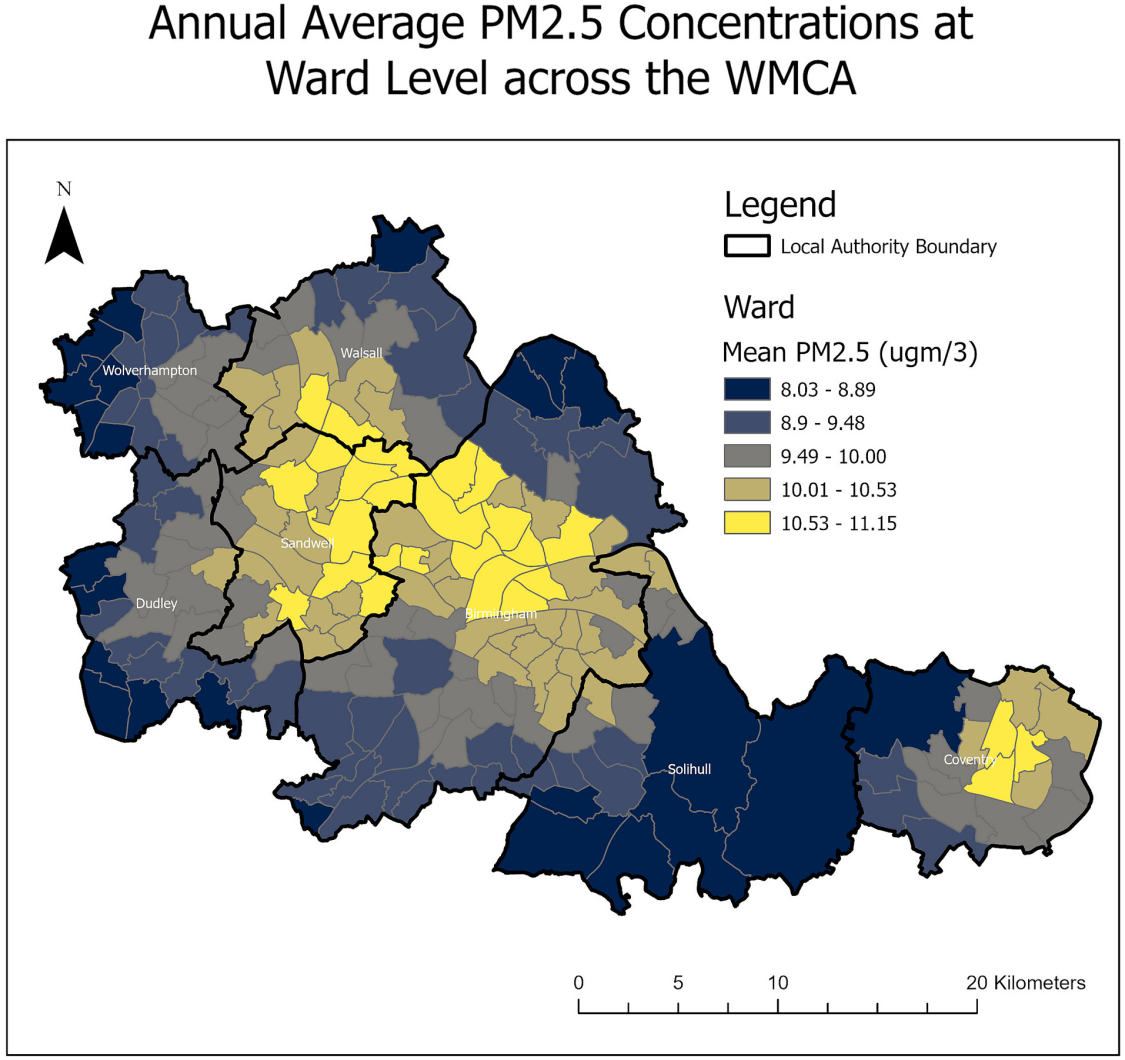
The WMCA study area showing the seven local authorities and associated wards, along with the 2019 mean PM_2.5_ concentrations.

In the following discussion, PM_2.5_ concentrations refer to ward-level average annual mean estimates, as outlined above. Actual concentrations vary with precise location within each ward, with some areas experiencing higher levels and some experiencing lower levels. The overall annual average PM_2.5_ concentration in the WMCA area was 9.7 μg/m^3^ in 2019, with a range from 8.0 to 11.2 μg/m^3^. There was variation across wards, from a maximum of 12.2 μg/m^3^ in Foleshill, Coventry, to a minimum of 7.7 μg/m^3^ in Tettenhall, Wolverhampton. Across all 192 wards within the WMCA area, 72 (37.5%) exceeded an annual average PM_2.5_ concentration of 10 μg/m^3^ (Limit Value/WHO AGQ interim target 4) in 2019 (Figure 1). These wards comprise a population of 1,197,119 individuals or 40.9% of the total WMCA population. Wards with the highest PM_2.5_ concentrations were in central Birmingham, Sandwell, south-central Walsall and Coventry local authority areas. Wolverhampton was the only local authority within the WMCA to have no wards with a mean PM_2.5_ concentration exceeding 10 μg/m^3^.

All wards in the study area exceeded the WHO AQGs for PM_2.5_ of 5 μg/m^3^ in 2019.

Table 3 shows the number and percentage of wards in each IMD decile exceeding 10 μg/m^3^ across the WMCA. The majority of wards in the more deprived IMD deciles (1–5) exceeded the limit value of 10 μg/m^3^, while only a minority of wards in the least deprived deciles (6–10) exceeded the limit value.

**Table 3:** The number of wards in each IMD decile exceeding 10 μg/m^3^ across the WMCA.

IMD Decile	1	2	3	4	5	6	7	8	9	10
Wards >10 μg/m^3^	15 (79%)	12 (63%)	10 (53%)	11 (58%)	6 (32%)	7 (37%)	6 (32%)	3 (16%)	2 (11%)	0 (0%)

‘1’ is the most deprived and ‘10’ is the least deprived. All deciles comprise 19 wards, apart from the 10^th^ decile, which has 21.

### 3.2 PWEL reductions

**Figure 2:**
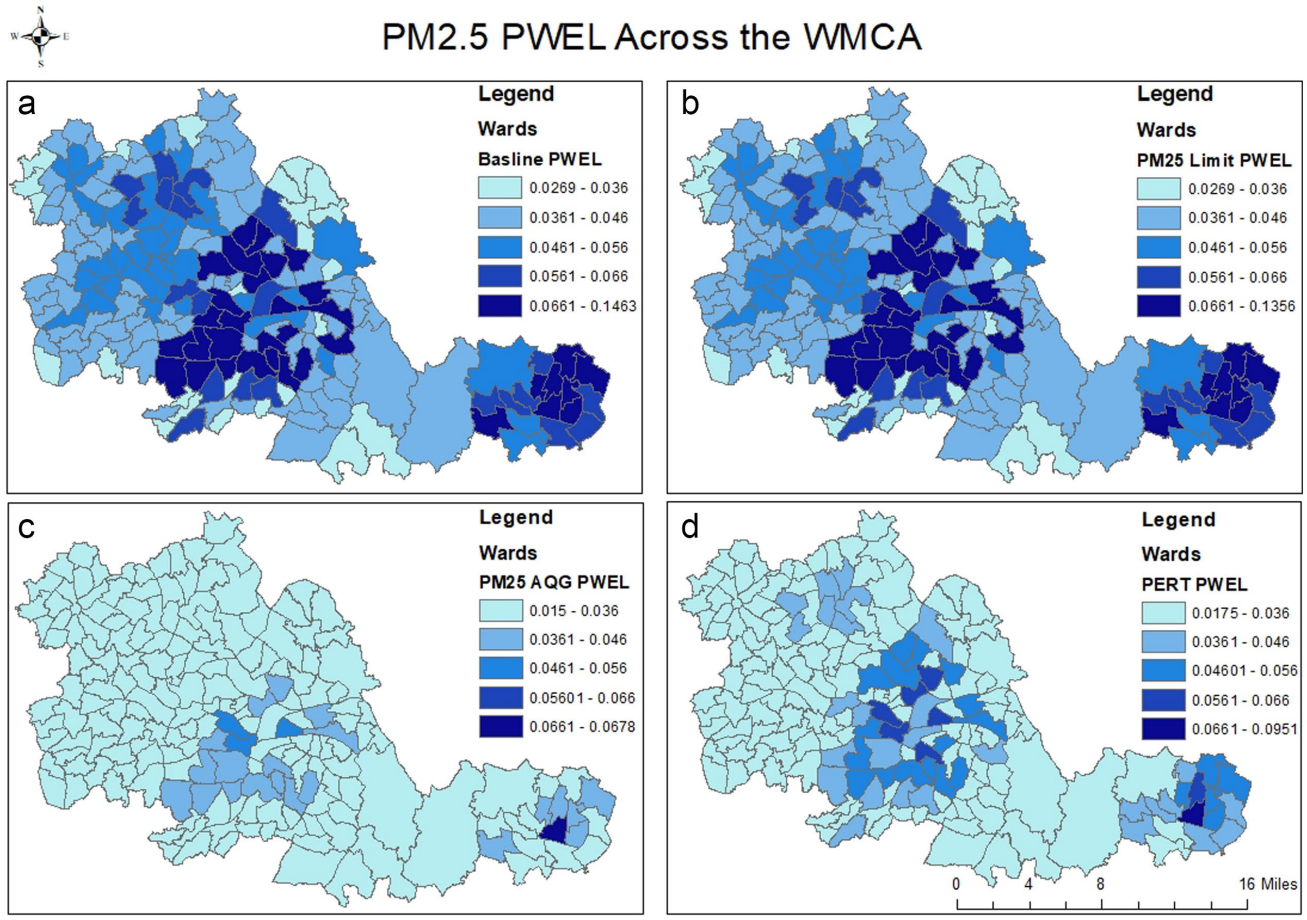
Changes in the PWEL across the WMCA from the 2019 Baseline conditions **(a)** to Scenario A (Limit Value) **(b)**, Scenario B (PERT) **(d)** and Scenario C (WHO) **(c)**. Note the change in scale for the first and final values across the three scenarios. Darker blue shades indicate a greater PWEL value.

Applying the selected scenarios (A-C), changes in the PWEL were calculated across the region, as shown in Figure 2.

#### 3.2.1 Scenario A (limit value)

Reductions in the PWEL from the baseline were small and focused on wards where levels exceeded 10 μg/m^3^, and they consequently saw a reduction in pollution concentrations, mostly within central city areas. These were calculated to benefit 1,197,119 people within the 72 wards. Only the highest values of the calculated PWEL were reduced, from 0.15 μg/m^3^ to 0.14 μg/m^3^, and changes were not large enough to visually alter the map representation of exposure values by lowering exceeding wards down the PWEL scale.

#### 3.2.2 Scenario B (PERT)

Achieving the PERT of a concentration reduction of 35% greatly reduced the PWEL across the region, to a greater extent than the Limit Value target compliance considered in scenario A (Figure 2). The large population centres of Birmingham and Coventry had PWEL values outside the lowest value range, albeit to a larger extent than in Scenario C. In addition, less PWEL reduction was observed in Wolverhampton.

#### 3.2.3 Scenario C (WHO)

Achieving the WHO AQG target of 5 μg/m^3^ influenced wards across the WMCA region and lowered the largest and smallest PWEL values (Figure 2). Only a small number of wards in central Birmingham and Coventry were outside the lowest PWEL values, due to the large populations found in these areas.

### 3.3 Air quality concentrations and ward-level deprivation

Under both the baseline conditions and the three scenarios, residents of wards with the highest IMD indices experienced the highest mean PM_2.5_ concentrations. These are generally located in the centre of the local authority footprints, notably Sandwell, Birmingham and Coventry. These findings are consistent with previous research highlighting the spatial patterns of urban deprivation and air pollution, although this is not ubiquitous across cities (19, 20). In addition, Birmingham and Coventry are the two most populous wards in the region, with Sandwell being the fourth behind Dudley. This population density also resulted in Birmingham, Coventry and Sandwell having the highest PWEL values in the WMCA area.

**Figure 3:**
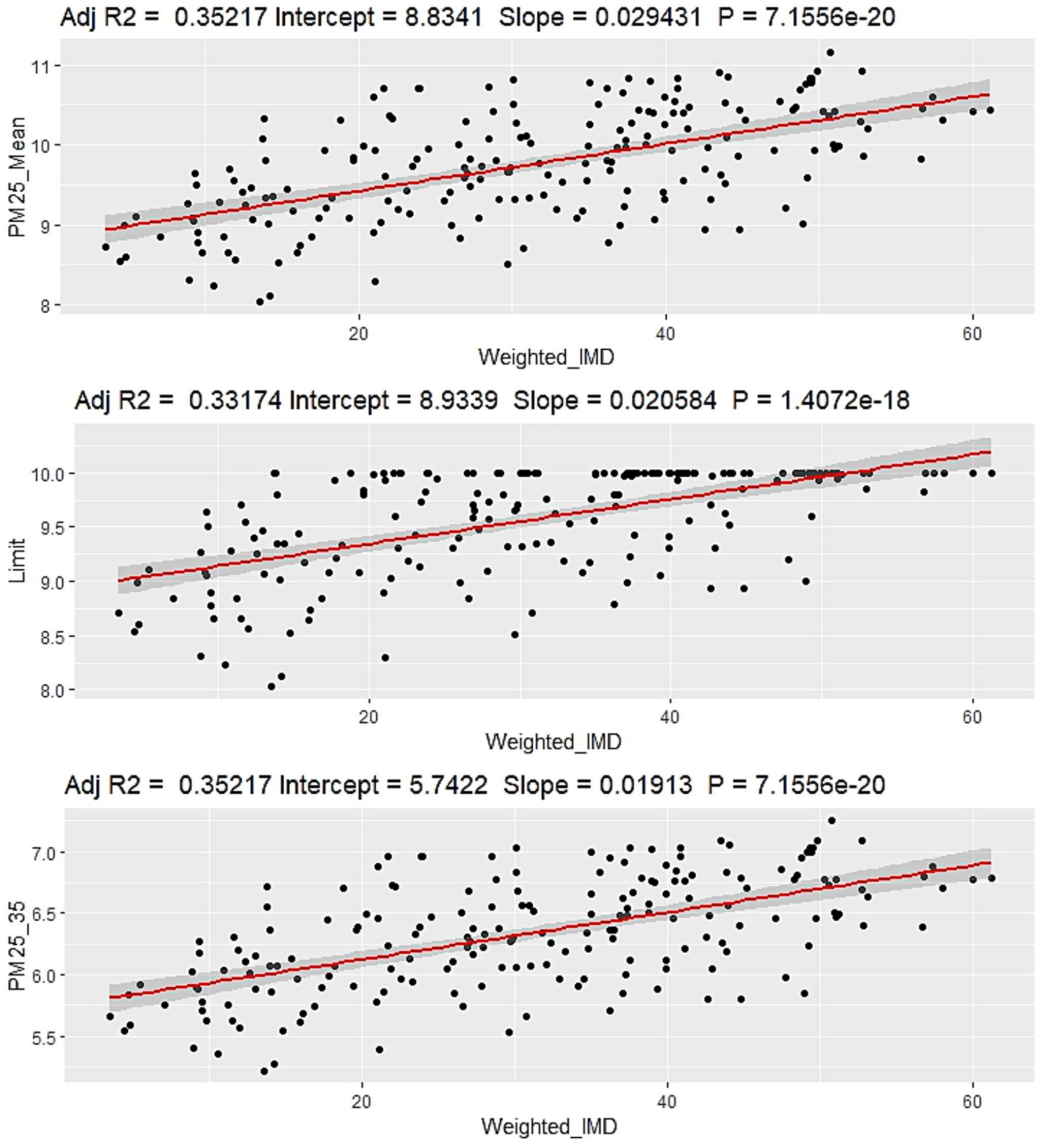
Relationships between the IMD score and PM_2.5_ concentration. Top: baseline concentrations (2019). Middle: Scenario A. Bottom: Scenario B. Scenario C is not shown as all values are fixed at 5 μg/m^3^, and therefore no relationship with IMD scores would be observed.

The more deprived wards in the WMCA area experienced the highest PM_2.5_ concentrations and PWEL figures under the baseline scenario (Figure 3). Under Scenario A, the most deprived wards would experience the greatest benefit if compliance with the Limit Value were achieved. In addition, under Scenario B (PERT), the most deprived wards would also benefit the most with regard to reduced absolute and relative concentrations, reflecting reductions in these areas of higher population density and that they exceed the threshold concentration target of 10 μg/ m^3^ by less than 35%.

Scenarios A, B and C all showed a reduction in PWEL values because of lower pollution values compared to the baseline conditions. Generally, lower IMD value wards have lower PWEL levels, although this is not uniform across the WMCA. In addition, the seven local authorities were ranked by their air quality concentrations. This showed that PM_2.5_ concentrations were lowest in Wolverhampton and highest in Sandwell (Table 4). However, Birmingham has the highest population and therefore the largest PWEL value. Consequently, Birmingham, Sandwell and Coventry—the second most populous local authority— would experience the greatest population-level benefit from region-wide reduced concentrations.

**Table 4:** Mean PM_2.5_ concentrations and population-weighted exposure levels for the seven local authorities that make up the WMCA study area.

Local authority	Wards >10 μg/ m^3^	Mean PM_2.5_ (μg/ m^3^)—Baseline	Mean PM_2.5_ (μg/ m^3^)—Scenario A	Mean PM_2.5_ (μg/ m^3^)—Scenario B	Mean PM_2.5_ (μg/ m^3^)—Scenario C	PWEL—Baseline	PWEL (μg/ m^3^)—Scenario A	PWEL (μg/ m^3^)—Scenario B	PWEL (μg/ m^3^)—Scenario C
Birmingham	33	9.91	9.68	6.44	5	9.81	9.62	6.38	0.028
Coventry	7	9.88	9.69	6.42	5	1.27	1.23	0.82	0.634
Dudley	1	9.29	9.28	6.04	5	1.02	1.02	0.67	0.023
Sandwell	20	10.39	9.97	6.75	5	1.17	1.12	0.76	0.023
Solihull	3	9.3	9.24	6.04	5	0.69	0.68	0.45	0.369
Walsall	8	9.87	9.7	6.41	5	0.97	0.95	0.63	0.024
Wolverhampton	0	9.1	9.1	5.92	5	0.82	0.82	0.53	0.022

## 4 Discussion

Of the 192 wards, 72 in the WMCA exceeded the Threshold Air Quality Target (WHO interim target / 2040 objective) of 10 μg/m^3^ for PM_2.5_ in the baseline scenario. Most of these wards are in central Birmingham, Sandwell and south-central Walsall, where higher levels of local pollution sources and traffic pollution are present (32). The majority are also the more deprived wards in the combined authority, with 54 of the wards exceeding the 10 ug/m^3^ level corresponding to the IMD deciles 1–5. For the PWEL, the same wards, especially in Birmingham, showed the highest values due to the large population clusters in the city centres.

Scenario A only reduced the PWEL in central areas with threshold exceedances, while Scenarios B and C had wider impacts across the region. Meeting the WHO AQG target greatly reduced the PWEL of the WMCA, as expected; however, it is suggested that the attainment of the AQG of an annual average PM_2.5_ concentration of 5 μg/m^3^ could be difficult or even implausible for the majority of developed urban areas in the UK, due to contributions from natural sources, transboundary pollution, and regional PM concentrations (33). Therefore, achieving the 35% PERT target may be more realistic and would have population benefits across a wider spatial scale of the WMCA. Furthermore, current air quality initiatives have not been viewed as public health actions, while the introduction of the PERT approach shifts the emphasis and will require additional consideration by policymakers. As a result of this analysis, and in line with the COMEAP report (2022), the PERT approach (scenario B) will likely be important in maximising broader public health benefits across the population. It would also mitigate the potential of widening inequalities in terms of air quality exposure that might occur with the implementation of a threshold target (22). For example, our scenarios suggest a marginal relationship between the IMD and air quality for Scenario A and Scenario B (0.021 vs. 0.019), but at this level of variation, *in situ* analysis would be needed to determine if there were significant differences.

Focused air quality improvements for the majority of deprived areas or local authorities with the highest PWEL values and the greatest number of wards exceeding targets may result in disproportionate benefits—that is, improvements in the number of wards exceeding targets but overall lower population health improvements. Reducing PM_2.5_ concentrations in specific wards (Scenario A) will be difficult due to the substantial contribution of regional pollution to PM_2.5_ levels and will have less benefit to the wider WMCA (34, 35). However, reducing domestic combustion could reduce PM2.5 pollution by 13.4% at a local level (35). Furthermore, recent monitoring and analysis of PM_2.5_ sources in the West Midlands show that biomass burning within the region makes up 25% of PM_2.5_ mass and is seven times higher than the levels in 2008–2010 (36). Similarly, the WMCA Air Quality Framework (25) highlights several public engagement policy options that aim to improve local air quality and reduce public exposure, which could be targeted at more deprived areas in the region:

- Reducing domestic solid fuel burning
- Supporting active travel and reducing car use
- Provide better information to support local decision-making to reduce exposure to air pollution

Consequently, to achieve improvements in the WMCA, regional actions to improve local air quality must complement local measures and align with national policy strategies. This is particularly true for PM emissions, which arise from a broad range of sources and, as shown in Figure 1, exert a substantial impact on local authorities and urban areas for attaining the WHO AQGs, with ~41% of the region exceeding interim target 4. Schemes that may be considered to help achieve the proposed hypothetical scenario air quality levels include the implementation of Low Traffic Neighbourhoods (LTNs), which have been successfully implemented in several metropolitan locations within the UK. The results from Birmingham and London have demonstrated reductions in PM and other pollutants detrimental to health, albeit with mixed community reception (37, 38). In addition, Clean Air Zones (or Ultra Low Emission Zones) have already been implemented in Birmingham and other cities, with expansions and modifications underway in some cities, notably London (39). These have had a generally positive impact on reducing NO_2_ levels but have shown mixed results for PM_2.5_. In addition, some studies suggest that they can divert non-compliant vehicles into more deprived neighbourhoods, which ultimately worsens air quality inequities—an issue this research aims to address (40, 41).

Future research may examine the potential impacts on disease morbidity, work productivity and wider societal costs (24), which could be calculated as part of the benefits gained from the reduction in the PWEL across the WMCA. In addition, research could explore intersectionality with age, gender and ethnicity as dimensions of inequality. This is particularly important as current mortality estimates are based on individuals over 30, while the WMCA has a younger-than-average population. This would also remove the current study’s limitation of not considering population changes over the time it would take for policies to reduce air pollution to the targeted scenario levels. A similar analysis, utilising the technique developed by Hall et al. (24), could also determine if wards have a higher risk exposure level due to pre-existing cardiorespiratory illnesses or age-related risk factors. Furthermore, working towards and achieving either the air quality threshold or the PERT will result in different spatial patterns of exposure and population impacts. The former approach is likely to leave significant variation in population exposure across the WMCA, particularly in Birmingham and Solihull. In contrast, the PERT would reduce the disparity between the highest and lowest exposure values, albeit with some central Birmingham and Coventry areas still at higher values than the rest of the region. Both scenarios would require additional research to determine the extent to which exposure has been reduced and to further reduce potential inequalities in the region.

## 5 Conclusion

To address the effects of transboundary, regional, and natural PM_2.5_ sources, regional actions to improve local air quality should be coordinated to complement local measures and utilise national policy strategies. This study utilised a PWEL metric to assess the impact of future changes in PM_2.5_ on a typical metropolitan location, in conjunction with IMD and population data. Approximately 41% of the WMCA population live in areas with PM_2.5_ levels over the WHO interim target 4 value of 10 μg/m^3^. This also introduces a bias towards the most deprived wards in the region. The region has a younger-than-average population that is more susceptible to adverse health effects, which compounds these issues. Three policy scenarios are presented for reducing future pollution concentrations (the 10 μg/m^3^ limit, PERT and WHO targets)—all of which would result in improved PWEL values and reduced exposure. The analysis of approaches for achieving either the 2040 Threshold Target or PERT showed that both would have population benefits of reducing exposure and associated health impacts. Of these, the PERT would provide broader population-level benefits. The threshold approach would reduce inequalities but provide fewer population-level benefits. This highlights the complexity of air quality improvement policies in metropolitan areas and the need for multiple strategies to achieve the best environmental and public health outcomes for policy decision-makers.

## Data Availability

The original contributions presented in the study are included in the article/supplementary material, further inquiries can be directed to the corresponding author.
